# Erector Spinae Plane (ESP) Block for Postoperative Pain Management after Open Oncologic Abdominal Surgery

**DOI:** 10.1155/2023/9010753

**Published:** 2023-06-15

**Authors:** Michael Dubilet, Benjamin F. Gruenbaum, Michael Semyonov, Shlomo Yaron Ishay, Anton Osyntsov, Michael Friger, Alexander Geftler, Alexander Zlotnik, Evgeni Brotfain

**Affiliations:** ^1^Department of Anesthesiology and Critical Care, General Intensive Care Unit, Soroka Medical Center, Faculty of Health Science, Ben-Gurion University of the Negev, Beer Sheva, Israel; ^2^Department of Anesthesiology and Perioperative Medicine, Mayo Clinic, Jacksonville, FL, USA; ^3^Department of Cardiothoracic Surgery, Soroka Medical Center, Faculty of Health Science, Ben-Gurion University of the Negev, Beer Sheva, Israel; ^4^Department of General Surgery B, Soroka Medical Center, Faculty of Health Science, Ben-Gurion University of the Negev, Beer Sheva, Israel; ^5^Department of Public Health, Faculty of Health Sciences, Ben-Gurion University of the Negev, Beer Sheva, Israel; ^6^Department of Orthopedic Surgery, Soroka Medical Center, Faculty of Health Science, Ben-Gurion University of the Negev, Beer Sheva, Israel

## Abstract

Patients undergoing abdominal oncologic surgical procedures require particular surgical and anesthesiologic considerations. Traditional pain management, such as opiate treatment, continuous epidural analgesia, and non-opioid drugs, may have serious side effects in this patient population. We evaluated erector spinae plane (ESP) blocks for postoperative pain management following elective oncologic abdominal surgeries. In this single-center, prospective, and randomized study, we recruited 100 patients who underwent elective oncological abdominal surgery between December 2020 and January 2022 at Soroka University Medical Center in Beer Sheva, Israel. We compared postoperative pain levels in patients who were treated with a preincisional ESP block in addition to traditional pain management with intravenous opioids, non-steroidal anti-inflammatory drugs (NSAIDs), and acetaminophen, compared to patients who were only given traditional pain management (control). Patients who were treated with a preincisional ESP block demonstrated significantly lower Visual Analog Scale scores at 60 minutes and 4, 8, and 12 hours following the surgery, compared to the control group (*p* < 0.001). Accordingly, patients in the ESP group required less morphine from 60 minutes to 12 hours after surgery, but they required increased non-opioid postoperative analgesia management at 4, 8, and 12 hours after surgery (*p* from 0.002 to <0.001) compared to the control group. In this study, we found ESP blocks to be a safe, technically simple, and effective treatment for postoperative pain management after elective oncologic abdominal procedures.

## 1. Introduction

Regional anesthesia complements and enhances multimodal analgesia for different types of abdominal surgery, with its role increasingly recognized [1], and may indeed alter outcomes [2–4]. In the past several years, more strategies have been developed for regional anesthetic approaches for abdominal surgery. Fascial plane blocks have become a recent focus as a potential replacement for thoracic paravertebral blocks, the current standard of care [5, 6]. Patients undergoing abdominal surgical procedures involving the integrity of the abdominal wall always require unique surgical and anesthetic considerations, in large part due to the severe pain that most patients report after abdominal surgery. These surgeries can be associated with serious complications including respiratory failure due to splinting, unproductive coughing of secretions and resultant pneumonia, and the development of the chronic pain condition known as the postlaparotomy pain syndrome [7].

However, traditional pain management in these patients, including opiate treatment, continuous epidural analgesia, and non-opioid drugs, may have serious side effects [8]. Opiates given at large doses can result in cough reflex and respiratory depression, requiring re-intubation and re-ventilation [9]. Thoracic epidural analgesia, though considered paramount compared to other analgesic options, requires significant clinical experience. Non-steroidal anti-inflammatory drugs (NSAIDs) and tramadol are weak analgesics inadequate for severe pain control and might be responsible for gastrointestinal bleeding [10].

The erector spinae plane (ESP) block is one of the newest techniques to be explored [11]. It was first described by Forero et al. in 2016 for the treatment of chronic thoracic neuropathic pain and postoperative pain in thoracic surgery [12]. It has also been shown to have suitable analgesic effects at the cervical, thoracic, and abdominal levels [13–15]. In addition, studies suggest that it provides effective analgesia in the upper or lower limbs if it is performed at the high thoracic and lumbar levels, respectively [13–15]. Additionally, it has a low rate of reported complications [16].

In this prospective study, we performed ESP blocks for postoperative pain management for different types of elective oncologic abdominal surgeries. We compared two groups of patients to evaluate the effects of incorporating a preincisional ESP block alongside traditional pain management using intravenous opioids and nonsteroidal anti-inflammatory drugs (NSAIDs). Specifically, we examined the postoperative pain level and incidence of postoperative nausea and vomiting in these groups. One group received both the ESP block and traditional pain management, while the other (control) group received only traditional pain management.

## 2. Patients and Methods

### 2.1. Patient Population

This was a single-center, prospective, and randomized study. This study was approved by the Human Research and Ethics Committee at Soroka Medical Center (RN-0355-19-SOR). In this study, we recruited all patients who underwent elective open oncological abdominal surgery between December 2020 and January 2022 at Soroka University Medical Center, a 1000-bed tertiary-care, level I trauma center and university teaching hospital located in Beer Sheva, Israel. Formal informed consent for the study was obtained on the day before surgery. One hundred patients were included. The study was registered with ClinicalTrials.gov identifier: NCT05512897.

### 2.2. Informed Consent

Informed consent was obtained from participants during the preoperative assessment. For eligible patients who refused to participate in the present study, the standard pain treatment was prescribed.

### 2.3. Inclusion Criteria

We recruited all patients older than 18 years of age, with an ASA physical status class between I and II, who were undergoing open oncologic abdominal operations (cancer of upper and low gastrointestinal tract).

### 2.4. Exclusion Criteria

Unconscious or mentally incompetent patients or those who refused to participate in the study were excluded.

### 2.5. Variables and Measurements

We collected data from electronic records during the patients' hospital and intensive care unit admission, including demographics of gender, age, underlying medical conditions, and admission diagnosis; Visual Analog Scale (VAS) levels; postoperative opioid and NSAIDs requirements; postoperative incidences of nausea and vomiting and other complications; type of surgery; and length of hospital stay.

### 2.6. Erector Spinae Plane (ESP) Block Technique

**Figure 1:**
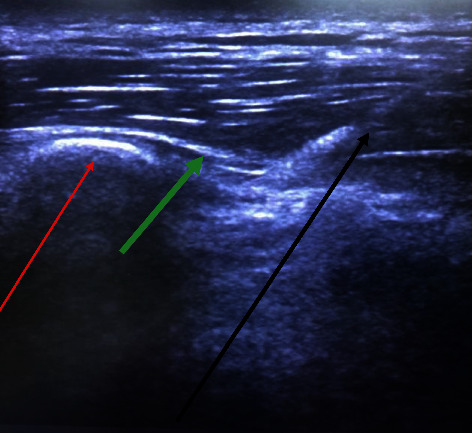
Ultrasound-guided ESP block (T5 level). Red line: transverse process of the T5 thoracic vertebra. Green line: erector spinae muscle. Black line: needle with local anesthetic spraying.

For this study, we performed the bilateral ESP block technique, which involves the patient sitting with support from a member of the care team or lying in a lateral decubitus position. Before each case, we did a preliminary scan with a linear ultrasound transducer (6–13 MHz) placed vertically on the patient's upper back, 2.5–3 cm lateral to midline. For sensory blockade of the appropriate dermatome, blocks were administered at levels T6–T10 for upper abdominal surgery and levels T8–L2 for lower abdominal incisions. The midline (spinous processes) and transverse processes were then marked bilaterally. Using a sterile technique, an 80 mm 22 G needle was inserted in cranial-caudal direction in-plane to the ultrasound transducer towards the transverse process. The needle crosses three layers of muscle, from posterior to anterior: trapezius, rhomboid, and erector spinae, until it touches the transverse process (Figure 1). Local anesthetic was injected via small increments for a total of 20–40 mL after careful repetitive aspiration to avoid intravascular injection. Either 0.2% ropivacaine or 0.375%–0.5% bupivacaine was used.

### 2.7. Study Protocol

The recruitment of patients was performed at the preoperative clinic visit as part of the first anesthesiology evaluation and preanesthesia examination. Goals of the study and the experimental protocol were explained to the patients along with the possible complications and adverse effects. Informed consent to be enrolled in the study and consent to be randomized into two groups were obtained at the same meeting. Randomization of the participants into two groups was performed using https://randomization.com.

**Figure 2:**
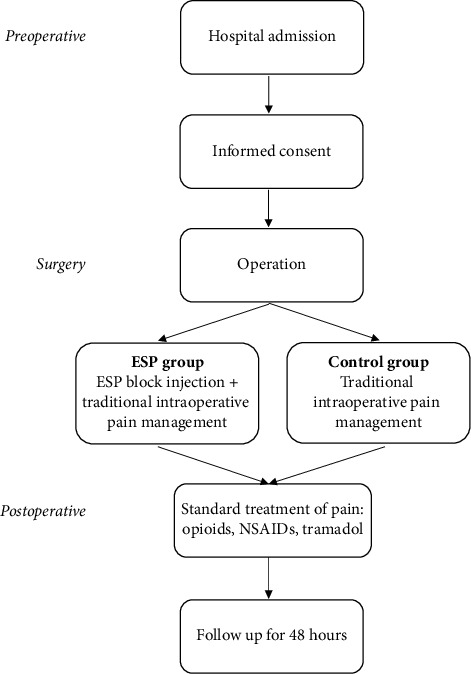
Study protocol.

We divided all patients into two groups before the beginning of the surgery (Figure 2). The control group received standard pain control treatment. The ESP study group received ESP block in addition to standard pain control treatment. Both study group patients were blinded in this study, meaning that they were not aware of which treatment was given to their respective group. The ESP block was performed after induction of general anesthesia and before the surgical incision. At the end of the surgery, after the patient was transferred to the postanesthesia care unit (PACU), vital signs and pain level were continuously monitored. During the postoperative period, the pain level of each patient was assessed with the VAS of pain (from 0–10, 0 = no pain, 10 = worst pain) by interviewing the patients at PACU admission. Following discharge from the PACU, VAS was obtained from these patients in the general surgery ward at time intervals of 4, 8, 12, 24, and 48 hours. While on the general surgery ward, patients received additional non-opioid postoperative analgesia management, including 125 mg intravenous tramadol and 1.25 g PO metamizole.

### 2.8. Standard Pain Control Treatment and Postoperative Nausea and Vomiting Assessment

Control and ESP patients received standard pain control that included treatment with opioids, NSAIDs, and acetaminophen (paracetamol) [17]. Postoperative pain was managed by 5–10 mg of intravenous morphine to achieve a VAS scale of none or mild pain (0–3) [18]. We continued to record events of postoperative nausea and vomiting within 48 hours after procedure.

### 2.9. Primary and Secondary Outcomes

Our primary outcomes were VAS pain scores at 1, 4, 8, 12, 24, and 48 hours postoperative and opioid analgesic requirements during the first 48 hours of the postoperative period. Secondary outcomes included nausea and vomiting incidence, non-opioid analgesic requirements, and variations in hemodynamic parameters.

### 2.10. Statistical Analysis

Assuming an annual admission rate of 500 patients during 1 year of the study enrollment, we planned to achieve a sample size of at least 100 patients. Sample size was calculated for the difference in postoperative cytokine levels. A total of 100 patients were needed for a significance level of 0.05 and a power of 90%. Each study group consisted of 50 patients for a total of 100 patients. To analyze our quantitative variables, we used the Kolmogorov–Smirnov test to determine their distribution. After conducting the test, we found that only age was distributed normally. As a result, we used the *t*-test to analyze age. For the other quantitative variables that were not normally distributed, we used the Mann–Whitney *U* test and chi-squared test.

The quantitative variables are presented by mean and standard deviation and/or median and interquartile range (IQR). The qualitative variables are presented by their distribution by percentage. For multivariate analysis, quantile regression was applied because dependent variables are not normally distributed. We performed quantile regression for the following quantiles: 0.1, 0.25, 0.5, 0.75, and 0.9. *p* values < 0.05 are considered as statistically significant. For statistical analysis, SPSS software version 26 (IBM) was applied.

## 3. Results

**Figure 3:**
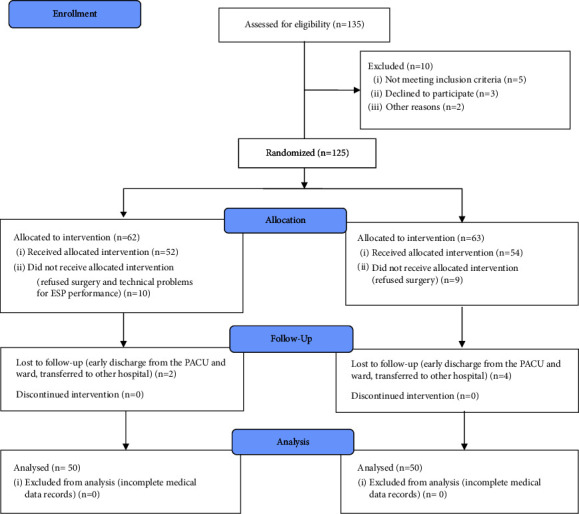
Consort flow diagram of present study.

After enrollment and the processes of randomization and exclusion, a total of 100 patients who underwent upper and low gastrointestinal tract oncologic open abdominal surgery in our institution were included in the study (Figure 3). The patients were randomly divided into 2 groups. One group of 50 patients was treated with the standard pain control treatment, and the other group of 50 patients received an ESP block before surgery started in addition to the standard pain control treatment.

Demographic data (age, gender, admission diagnosis, underlying medical conditions, and type of gastrointestinal surgery) are summarized in Table 1. There was no statistically significant difference in any of the demographic characteristics, admission diagnosis, or the type of surgery between the study groups. There was a prevalence of comorbidities of chronic ischemic heart disease (CIHD) and chronic obstructive pulmonary disease (COPD) in the control group compared to the ESP group (Table 1).

Table 2 describes the postoperative hemodynamic parameters in the two groups in the PACU and in the general surgery unit. Patients who were treated with a preincisional ESP block in addition to standard pain control treatment after surgery presented significantly lower heart rate and systolic and diastolic blood pressure parameters compared to the control group (*p* from 0.03 to <0.001, see Table 2).

Table 3 demonstrates the length of hospital stay, postoperative pain levels (VAS scores), and opioid and non-opioid analgesic requirements of both study groups. Patients who were treated with a preincisional ESP block demonstrated significantly lower VAS scores at 60 minutes and 4, 8, and 12 hours following the surgery, compared to control group patients (*p* < 0.001, see Table 3). In contrast, at 24 and 48 hours following surgery, VAS score levels were found to be significantly lower in patients in the control compared to the ESP group (*p* < 0.001 and 0.01, Table 3).

Accordingly, patients in the ESP group required fewer postoperative opioids (morphine) at 60 minutes to 12 hours after surgery and required additional non-opioid analgesia management at 4, 8, and 12 hours after surgery (*p* from 0.002 to <0.001, Table 3) compared to the control group. Similarly, at 24 and 48 hours following surgery, the use of opioids and additional non-opioid postoperative analgesia was lower in the control group than in the ESP group (*p*=0.002 and < 0.001, respectively, Table 3).

Table 4 shows the occurrence of postoperative nausea and vomiting from patient admission to PACU until 48 hours following surgery. The overall incidence of postoperative nausea and vomiting was significantly higher in the control group compared to the ESP group (*p* from 0.001 to <0.001, Table 4) at admission to PACU and at 60 minutes and 4, 8, and 12 hours after surgery.

We also analyzed predicting factors of adequate postoperative analgesia level (defined as VAS score less than 5) on the VAS score at 60 minutes after admission to the PACU by multivariate statistical analysis. Since VAS scores within the study population were non-normally distributed outcomes, the data were analyzed using quantile regression at quantiles 0.1, 0.25, 0.5, 0.75, and 0.9. Based on the results of quantile regression analysis, patients in the ESP group were considered a significantly relevant predictor with negative regression coefficients for Q10, Q25, Q50, Q75, and Q90 (−2.478CHF(critical heat flux), *p* − 0.0015; −3.8CHF, *p* < 0.0001; −10.2CHF, *p* < 0.0001; −3.8CHF, *p* < 0.0001; and −2.47CHF, *p* − 0.015, respectively) for postoperative VAS score (at 60 minutes after surgery). Male gender was found to have a positive regression coefficient only for Q10 (2.43 CHF, *p* − 0.017) for postoperative VAS score (at 60 minutes after surgery). In addition, admission diagnosis was found to have a positive regression coefficient for Q50, Q75, and Q90 (3.08CHF, *p* − 0.003 and 2.28CHF, *p* − 0.024, subsequently) for postoperative VAS score (at 60 minutes). None of the other parameters were found to have predictive value on postoperative VAS score (at 60 minutes).

## 4. Discussion

In this study, we performed ultrasound-guided bilateral preincision ESP blocks alongside the standard pain protocol for postoperative analgesia. Our results showed that study group patients who received the ESP block along with the standard protocol had better postoperative pain control, as indicated by lower VAS scores, less opioid usage, and lower incidence rates of postoperative nausea and vomiting compared to the control group, who only received the standard protocol. We did not observe any postoperative complications related to the ESP block, indicating that it is a safe and straightforward option for postoperative pain management. These findings are consistent with previously published literature.

Our analysis showed a reduction in postoperative VAS scores and opioid usage in both univariate and multivariate analyses. We chose VAS at 60 minutes and postoperative morphine use for our multivariate analysis, as they are the most effective indicators of analgesia. We observed that VAS at 60 minutes was not normally distributed among the study population. Therefore, we performed quantile regression analysis to identify independent risk factors that influence pain across different quantiles. The analysis revealed that the ESP group was a significant predictor of negative regression coefficients for Q10, Q25, Q50, Q75, and Q90 quantiles of postoperative VAS score (at 60 minutes), indicating its effectiveness in reducing pain across different levels. Additionally, male sex was found to be a positive regression coefficient only for Q10 of postoperative VAS score.

During the last decade, interest in using different types of nerve blocks for postoperative pain control after abdominal surgery has emerged [8, 19, 20]. There are noticeable limitations in the existing techniques for postoperative pain control for these patients. Paravertebral blockade requires advanced technical proficiency to perform properly. It also has side effects that are similar to epidural anesthesia or intercostal nerve block due to the large amounts of local anesthetic needed to achieve adequate postoperative pain control [21]. Thoracic epidural analgesia is a preferred approach for analgesia compared to systemic opioids which bear adverse effects [22–24]. However, despite its efficacy for analgesia, it has not been shown that thoracic epidural analgesia reduces length of hospital stay, incidence of ileus, or postoperative complications after open abdominal surgery [25]. In minimally invasive abdominal surgery, there are insufficient data to espouse the use of epidural analgesia in a multimodal approach.

Only several years ago, the ESP block was introduced as an alternative successful method for chronic and acute pain management in a variety of surgical and non-surgical patient populations [12]. This type of block can be administered as a single injection or via catheter placement for continuous infusion. The first report of the successful use of this procedure was described in 2016 by Forero and colleagues [12]. In that study, researchers administered the ESP block to address thoracic neuropathic pain in a patient with metastatic disease of the ribs and rib fractures [12, 26]. In addition, Forero et al. administered several ESP blocks at the T2/T3 level to manage chronic shoulder pain in an elderly male patient [27]. After the block, immediate and significant analgesia, along with improved range of motion, was noted.

Since these early studies, the ESP block has been used effectively in a variety of procedures including the Nuss procedure, thoracotomies, percutaneous nephrolithotomies, ventral hernia repairs, and lumbar fusion [26]. Even more, Elkoundi et al. reported successful usage of ESP blocks for hyperalgesic acute pancreatitis [28].

Bilateral ESP blocks have also been indicated for primary use in many upper and lower abdominal laparoscopic procedures, including laparoscopic sleeve gastrectomy, laparoscopic cholecystectomy, and inguinal hernia, in addition to endoscopic retrograde cholangiopancreatography, laparoscopic varicocelectomy, laparoscopic hepatic cystectomy, laparoscopic nephrectomy, laparoscopic hysterectomy, laparoscopic Nissen fundoplication, laparoscopic hysterectomy, laparoscopic ovarian cystectomy, and laparoscopic hemicolectomy [29]. For example, Chin et al. described four cases of preoperative bilateral ESP block for postoperative pain management after laparoscopic ventral hernia repair [30]. In all cases, adequate postoperative analgesia level was found. No complications have been observed after ESP block performance. There is also recently published literature, most of them case report series, about successful usage of ESP blocks for postoperative analgesia after open abdominal procedures [29].

With regard to gender, male sex had a positive regression coefficient for postoperative VAS score in this study, but with inconsistent findings of a correlation between gender and postoperative pain outcomes. In contrast to our findings, Cepeda and Carr [31] and Taenzer et al. [32] indicated strong positive correlation between female gender and postoperative analgesic consumption. However, Chang et al. failed to demonstrate any correlation between gender and postoperative analgesic requirements [33]. The relationship between gender and postoperative pain outcomes remains uncertain.

In our study, the admission diagnosis of low GI pathologies was found to have a positive regression coefficient for Q50, Q75, and Q90 for postoperative (at 60 minutes) VAS score. This finding is correlated well with previously published data that determine that abdominal surgery, especially for cancer, is a well-known positive predictor for postoperative analgesic consumption [34].

In the present study, the main analgesic effect of the ESP block lasted until 12 hours after surgery. At 24 hours after surgery, we demonstrated significantly increased opioid requirements in ESP group patients and found subsequently increased VAS scores in the same study group. The reasonable explanation of this result might be related to the phenomenon of “rebound pain” [35]. Rebound pain has been defined as an extremely severe pain that happens after a peripheral nerve block resolution when sensitivity returns [35, 36]. Although its overall incidence is not known, it seems to occur most often between 12 and 24 hours after surgery and it has a negative effect on sleep quality.

Multimodal approaches could mitigate the presentation of rebound pain, such as preemptive analgesia (before the effects of the block stop), intra-articular or intravenous anti-inflammatory medications, and use of adjuvants in nerve block solutions [17]. We suggest that clinicians educate their patients about the possibility of rebound pain so that they adhere to recommendations for preemptive analgesics and are prepared for postoperative pain management. Managing the effects of rebound pain through preemptive strategies and patient awareness is an important component of safe, effective regional anesthesia and the reduction of long-term opioid use and its resultant negative effects [36]. In our hospital, there is a standard protocol for postoperative pain management, including both opioid and non-opioid medications. However, in accordance with those findings about rebound pain after nerve block, our postoperative pain protocol and our patient education approach may need to be reassessed to ensure better analgesic clinical outcome after surgical procedures.

This study has several limitations, such as relying on data from only one medical center and its relatively small sample size. In addition, our data did not contain complete records of complications that may have taken place during or after intra-hospital transport of the patients following their discharge from the PACU. Also, there was some difference in the prevalence of comorbidities between the study groups that could have had an impact upon the outcomes observed. We anticipate that future larger scale prospective studies on this topic will correct these limitations.

## 5. Conclusion

Here, we describe an ESP block for postoperative pain control after abdominal oncologic procedures. We found ESP blocks to be safe, technically simple, and effective for postoperative pain management after elective oncologic abdominal procedures. However, in light of relatively high VAS score rates and opioid requirements observed at 24 hours after surgery, likely as a result of a rebound pain mechanism after nerve block, we advise an appropriate postoperative pain protocol and patient education to improve clinical outcome. This study contributes to the ongoing research into the implementation of ESP blocks as a viable pain treatment protocol, suggesting its efficacy but advising it as part of a comprehensive postoperative pain protocol.

## Figures and Tables

**Table 1:** Comparison of demographic characteristics, admission diagnosis, length of hospital stay, and types of surgery as mean ± SD, *n*, and %.

	Control group^*∗*^	ESP group^*∗*^	*P* value^*∗∗*^
	(*n* = 50)	(*n* = 50)	
Age, years (mean ± SD^a^)	64.1 ± 13.8	61.9 ± 17.8	0.505
Gender (male): no. (%)	38 (76%)	44 (88%)	0.063

*Underlying conditions*: *no. (%)*^*∗∗∗*^			
None	21 (42%)	25 (50%)	0.04
CIHD	9 (18%)	6 (12%)	0.04
COPD	6 (12%)	4 (8%)	0.03
DM II	5 (10%)	5 (10%)	0.08
HTN	4 (8%)	5 (12%)	0.1
Others^*∗∗∗*^	5 (12%)	5 (12%)	0.2

*Admission diagnosis*: *no. (%)*^*∗∗∗∗*^			
Upper GI pathologies	23 (46%)	22 (44%)	0.81
Low GI pathologies	27 (54%)	28 (56%)	0.8

*Type of surgery*: *no. (%)*^*∗∗∗∗∗*^			
Upper GI surgery	20 (40%)	21 (42%)	0.83
Low GI tract surgery	30 (60%)	29 (58%)	0.84

^*∗*^Control group: patients received standard pain control treatment; ESP group: patients received ESP block in addition to standard pain control treatment. ^*∗∗*^Data are considered statistically significant if *p* < 0.05. ^*∗∗∗*^Underlying conditions: CIHD—chronic ischemic heart disease; COPD—chronic obstructive pulmonary disease; DM II—diabetes mellitus type II; HTN—chronic hypertension; others include hypothyroidism, chronic peptic ulcer disease, and chronic renal failure. ^*∗∗∗∗*^Admission diagnosis: gastric or pancreatic cancer, and cancer of the colon, kidney, rectum, or adrenal. ^*∗∗∗∗∗*^Upper GI surgeries included gastrectomy or small bowel resections. Low GI tract surgeries include colectomy, hemicolectomy, and resection of rectum. ^**a**^SD: standard deviation.

**Table 2:** Comparison of hemodynamic parameters in the postoperative period as mean ± SD.

	Control group^*∗*^	ESP group^*∗*^	*P* value^*∗∗*^
	(*n* = 50)	(*n* = 50)	
*Heart rate (beats, mean* *±* *SD)*			
Admission to PACU	85.4 ± 9.7	73.52 ± 8.78	<0.001
At 60 min	87.12 ± 11.2	75.5 ± 9.5	<0.001
At 4 hours	85.48 ± 10.7	76.58 ± 9.01	<0.001
At 8 hours	86.2 ± 12.4	75.68 ± 8.5	0.001
At 12 hours	82.74 ± 10.05	74.42 ± 8.02	<0.001
At 24 hours	79.8 ± 9.4	79.14 ± 12.9	0.77
At 48 hours	75.06 ± 7.4	78.76 ± 8	0.04

*Systolic blood pressure (mmHg, mean* *±* *SD)*			
Admission to PACU	138.5 ± 16.37	129.16 ± 16.9	0.004
At 60 min	137.26 ± 23.5	126.6 ± 17.04	0.007
At 4 hours	137.1 ± 14.8	124.7 ± 15.3	<0.001
At 8 hours	135.8 ± 14.03	126.9 ± 13.02	0.001
At 12 hours	132.9 ± 14.6	125.9 ± 14.53	0.005
At 24 hours	134.1 ± 12.9	125.1 ± 14.6	<0.001
At 48 hours	128.1 ± 12.6	129.4 ± 17.4	0.62

*Diastolic blood pressure (mmHg, mean* *±* *SD)*			
Admission to PACU	74.44 ± 14.6	69.04 ± 14.12	0.004
At 60 min	74.54 ± 14.4	69.08 ± 11.57	0.087
At 4 hours	77.48 ± 11.65	71.7 ± 12.6	0.03
At 8 hours	77.8 ± 11.6	68.3 ± 10.6	<0.001
At 12 hours	75.38 ± 10.56	67.9 ± 7.9	<0.001
At 24 hours	68.2 ± 14.9	59.7 ± 14.1	0.01
At 48 hours	71.56 ± 11.4	69.9 ± 12.1	0.43

^*∗*^Control group: patients received standard pain control treatment; ESP group: patients received ESP block in addition to standard pain control treatment. ^*∗∗*^Data are considered statistically significant if *p* < 0.05.

**Table 3:** Comparison of perioperative outcomes as median (IQR).

	Control group^*∗*^	ESP group^*∗*^	*P* value^*∗∗*^
	(*n* = 50)	(*n* = 50)	
Length of hospital stay (days, median (IQR))	4 (3–5)	3 (2–5)	0.68

*VAS * ^*a*^ *score postoperative (mg, median (IQR))*			
An admission to PACU	0 (0)	0 (0)	0.15
At 60 min	2 (2-3)	1 (1-2)	<0.001
At 4 hours^b^	4 (3-5)	2 (1-3)	<0.001
At 8 hours	5 (4-5)	3 (2-3)	<0.001
At 12 hours	5 (4.75–5.25)	2 (2-3)	<0.001
At 24 hours	5 (4-5)	5 (5-6)	<0.001
At 48 hours	3 (3-4)	4 (3-4)	0.01

*Opioid postoperative treatment (mg, median (IQR))*			
An admission to PACU	10 (10-10)	10 (5–10)	0.002
At 60 min	10 (5–10)	5 (0–5)	<0.001
At 4 hours	1 (0–3)	0 (0)	<0.001
At 8 hours	1.5 (0–3)	0 (0–0.75)	<0.001
At 12 hours	0 (0–3)	0 (0)	0.0017
At 24 hours	0 (0–0.75)	1.5 (0–3)	0.002
At 48 hours	0 (0)	0 (0)	0.15

*Additional non-opioid postoperative analgesia * ^*c*^ *management (n, median (IQR))*			
At 4 hours	0 (0-1)	0 (0)	<0.001
At 8 hours	1 (0-1)	0 (0-1)	<0.001
At 12 hours	1 (0-1)	0 (0)	<0.001
At 24 hours	0 (0-1)	1 (0-1)	0.159
At 48 hours	1 (0-1)	0 (0)	<0.001

^*∗*^Control group: patients received standard pain control treatment; ESP group: patients received ESP block in addition to standard pain control treatment. ^*∗∗*^Data are considered statistically significant if *p* < 0.05. ^a^VAS: Visual Analog Scale for pain assessment. ^b^At the regular general surgery ward. ^c^Non-opioid postoperative analgesic management includes 125 mg IV tramadol + 1.25 g PO metamizole.

**Table 4:** Comparison of incidences of postoperative nausea and vomiting as *n* (%).

	Control group^*∗*^	ESP group^*∗*^	*P* value^*∗∗*^
	(*n* = 50) (%)	(*n* = 50) (%)	
Postoperative nausea and vomiting (*n* (%))			
An admission to PACU	27 (54)	11 (22)	0.001
At 60 min	26 (52)	7 (14)	<0.001
At 4 hours	30 (60)	13 (26)	0.001
At 8 hours	32 (64)	5 (10)	<0.001
At 12 hours	18 (36)	3 (6)	<0.001
At 24 hours	13 (26)	12 (24)	0.5
At 48 hours	22 (44)	15 (30)	0.1

^*∗*^Control group: patients received standard pain control treatment; ESP group: patients received ESP block in addition to standard pain control treatment. ^*∗∗*^Data are considered statistically significant if *p* < 0.05.

## Data Availability

The data used to support the findings of this study are available from the corresponding author upon reasonable request.

## Abbreviations

CHF: Critical heat flux
CIHD: Chronic ischemic heart disease
COPD: Chronic obstructive pulmonary disease
DM II: Diabetes mellitus type II
ESP: Erector spinae plane
GI: Gastrointestinal
HTN: Chronic hypertension
IQR: Interquartile range
NSAIDs: Non-steroidal anti-inflammatory drugs
PACU: Postanesthesia care unit
VAS: Visual Analog Scale.
